# Development and validation of interpretable machine learning models for predicting cumulative live birth rates in assisted reproductive technology: a SHAP-based analysis

**DOI:** 10.3389/fendo.2026.1949824

**Published:** 2026-10-07

**Authors:** XiaoQian Fu, Yisi Qin, Qianyi Huang, Huimei Wu, Li Jiang, Lidan Liu

**Affiliations:** Reproductive Medical Center, The First Affiliated Hospital of Guangxi Medical University, Nanning, China

**Keywords:** assisted reproductive technology, cumulative live birth rate, gradient boosting, machine learning, SHAP analysis

## Abstract

**Objective:**

To develop and validate interpretable machine-learning models predicting cumulative live birth rate (CLBR) in ART patients.

**Methods:**

In this single-center retrospective cohort (January 2019–June 2024), 7,773 oocyte retrieval cycles were analysed. XGBoost gain ranking, applied to all screened cycles with native handling of missing values, selected eight predictors that were complete in every cycle, so no cycle was excluded for missing data. Six algorithms were compared by stratified 10-fold cross-validation (n=5,441) and an independent test set (n=2,332). We further performed temporal validation (2019–2022 vs 2023–2024), benchmarked against a McLernon-style logistic regression and against a gradient-boosting model restricted to pre-treatment variables, and assessed age-stratified calibration, discrimination (AUC), decision-curve analysis (DCA), and SHAP interpretability.

**Results:**

Gradient boosting showed the best discrimination on the random-split test set (AUC 0.762; 95% CI, 0.743–0.781; Brier 0.185). Under temporal validation, performance remained robust (AUC 0.712; 95% CI, 0.686–0.737). The full model significantly exceeded the logistic-regression benchmark (0.761 vs 0.691; DeLong p<0.0001; NRI 0.629, 95% CI 0.550–0.712; IDI 0.105, 95% CI 0.090–0.121). However, a gradient-boosting model restricted to the same pre-treatment variables did not outperform that benchmark (0.693 vs 0.691; p=0.72), indicating that the gain was attributable to the inclusion of embryological information rather than to the learning algorithm itself. DCA demonstrated positive net benefit across thresholds 0.01–0.94. SHAP identified female age (mean |SHAP| 0.494) and embryo quality (0.451) as dominant predictors, with an inflection at 38–40 years.

**Conclusions:**

This interpretable framework delivers robust, well-calibrated CLBR prediction that remains stable under temporal validation while preserving transparency via SHAP. Its advantage over an established pre-treatment benchmark derives from incorporating embryological information rather than from the machine learning algorithm: precise estimation of cumulative live birth requires embryological data that become available only after oocyte retrieval and embryo development, rather than depending on which algorithm is used to model that information.

## Introduction

Assisted reproductive technology (ART) has revolutionized the treatment of infertility, with over 1 million cycles performed annually in Europe alone and an estimated 2–3 million cycles worldwide (1). Despite significant advances in clinical protocols and laboratory techniques, success rates remain highly variable, with cumulative live birth outcomes showing substantial heterogeneity across patient populations based on factors including maternal age, ovarian reserve, and treatment history (2, 3). This variability underscores the critical need for accurate prognostic tools to guide clinical decision-making, optimize resource allocation, and provide realistic expectations for patients undergoing fertility treatment (4, 5).

Cumulative live birth rate (CLBR), defined as the probability of achieving at least one live birth from a single oocyte retrieval cycle including all fresh and frozen embryo transfers, represents the most clinically meaningful outcome measure in ART (6). Unlike per-cycle pregnancy rates, CLBR reflects the true reproductive potential of a complete treatment episode and better informs patient counseling and shared decision-making (7). However, accurate prediction of CLBR remains challenging due to the complex interplay of multiple biological, clinical, and treatment-related factors that influence reproductive outcomes (8).

Traditional prediction models in reproductive medicine have predominantly relied on logistic regression and simple clinical scoring systems, which incorporate limited sets of variables such as female age, ovarian reserve markers, and previous treatment history (9). While these models provide valuable baseline estimates, they are constrained by assumptions of linear relationships and limited capacity to capture complex, non-linear interactions among predictive features (9). Recent systematic reviews have reported that existing CLBR prediction models demonstrate moderate discriminative performance (area under the curve [AUC] 0.63-0.68) and often lack clinical utility assessment (10).

Machine learning approaches offer potential advantages over conventional statistical methods by automatically identifying complex patterns, handling high-dimensional data, and modeling non-linear relationships without explicit specification (11). Several studies have explored machine learning for ART outcome prediction, demonstrating superior performance compared to traditional models (12). However, most investigations have focused on single-cycle pregnancy prediction rather than CLBR, employed limited feature sets (13), or lacked comprehensive model interpretability—a critical requirement for clinical adoption (14). The “black box” nature of many machine learning algorithms has hindered their integration into clinical practice, as clinicians require transparent, explainable predictions to inform treatment decisions and patient counseling (15).

Recent advances in explainable artificial intelligence, particularly SHapley Additive exPlanations (SHAP), provide model-agnostic frameworks for interpreting complex machine learning predictions at both global and individual levels (16). SHAP values quantify each feature’s contribution to individual predictions while maintaining consistency with established game-theoretic principles, enabling clinicians to understand not only what a model predicts but why (17). Despite these methodological advances, few studies have systematically applied interpretable machine learning frameworks to CLBR prediction with comprehensive evaluation of model calibration, clinical utility, and robustness (18).

This study aimed to develop and validate interpretable machine learning models for predicting cumulative live birth rates in ART patients, addressing key limitations of existing approaches. We systematically compared six machine learning algorithms using rigorous cross-validation and testing set evaluation, assessed model calibration and clinical decision-making utility through decision curve analysis, and employed SHAP-based interpretability analysis to provide transparent, clinically actionable explanations of model predictions.

## Materials and methods

### Study design and population

This retrospective cohort study aimed to develop and validate machine learning models for predicting cumulative live birth rates following assisted reproductive technology (ART). The study population consisted of patients who underwent ART cycles at the Guangxi Reproductive Medical Center, The First Affiliated Hospital of Guangxi Medical University, between January 2019 and June 2024. The unit of analysis was the oocyte retrieval cycle. The primary outcome was cumulative live birth, defined as the delivery of at least one live neonate resulting from a single oocyte retrieval cycle, encompassing all fresh and frozen embryo transfers associated with that cycle. Conservative cumulative live birth rate was calculated by assuming no live birth would occur for cycles with untransferred cryopreserved embryos remaining at the end of the observation period, thereby providing a minimum estimate of treatment success (4). The overall study design and analytic workflow are summarized in **Figure 1**.

**Figure 1:**
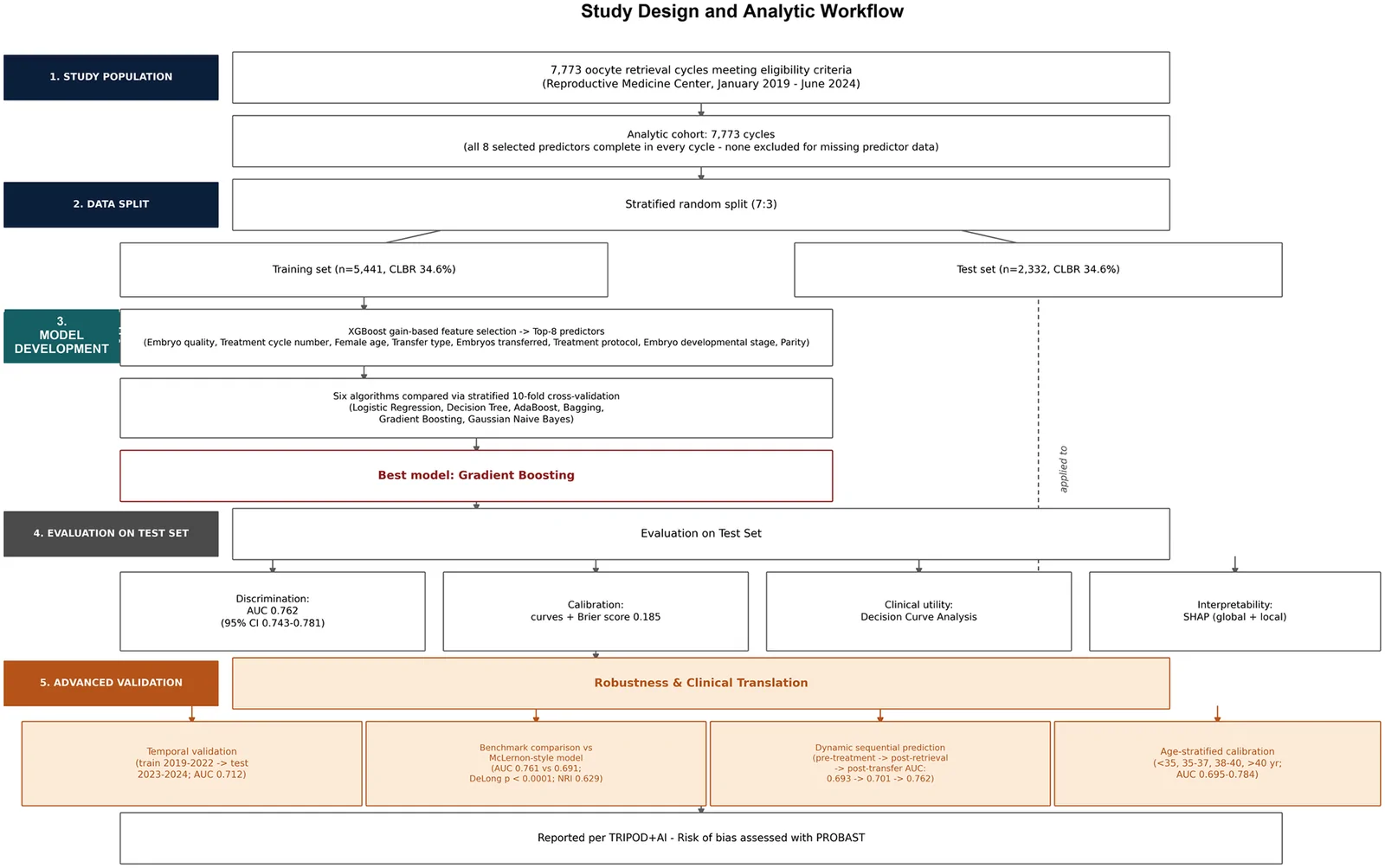
Study design and analytic workflow. Flow diagram summarizing the study population, data split, model development and selection, test-set evaluation, and advanced validation steps described in Materials and Methods.

Inclusion criteria comprised women who completed a single ART cycle with comprehensive baseline clinical data and outcome records; cycles without embryo transfer and cycles with undefined pregnancy outcomes were not eligible. The analytic population of 7,773 oocyte retrieval cycles reported throughout this study reflects cycles remaining after these eligibility criteria had already been applied at the point of data extraction from the clinical and embryology databases; because the pre-eligibility cycle log was not retained in the analytic dataset, the number of cycles excluded under each criterion cannot be reported separately (see Limitations). The study protocol was approved by the Ethics Committee of the First Affiliated Hospital of Guangxi Medical University.

### Data collection and variables

Clinical and laboratory data were extracted from electronic medical records and embryology databases. The following variables were collected: demographic characteristics (female age, male age, body mass index), hormonal parameters (anti-Müllerian hormone, luteinizing hormone, estradiol, progesterone), ovarian reserve markers (total follicle count, oocytes retrieved), treatment protocol details, embryological parameters (embryo quality, cell stage, number of embryos transferred), endometrial characteristics (thickness, pattern), and semen parameters (volume, density, viability, motility, morphology, measured both pre- and post-preparation).

Embryo quality was recorded as a five-level cycle-level composite variable that jointly encoded the number of embryos transferred and the number of high-quality embryos, according to the center’s embryology scoring rule. For single-embryo transfers, Quality 1 indicated that the transferred embryo was not classified as high quality, whereas Quality 3 indicated a high-quality embryo. For double-embryo transfers, Quality 2 indicated that neither embryo was high quality, Quality 4 indicated that one embryo was high quality, and Quality 5 indicated that both embryos were high quality. Thus, Quality 1-5 should not be interpreted as a universal per-embryo morphology grade. Embryo developmental stage was recorded separately as an independent predictor and was not incorporated into this five-level Quality variable.

Additional variables included parity, gravidity, abortion history, duration and type of infertility, abstinence duration, and semen source. Treatment protocol and cycle were recorded as two distinct indicators. The treatment protocol refers to the specific therapeutic strategy employed during the ART cycle, while the cycle was categorized into either a frozen embryo transfer (FET) cycle or a fresh embryo transfer (fresh ET) cycle. FET cycles involve the use of cryopreserved embryos, whereas fresh ET cycles involve the transfer of newly retrieved embryos. Continuous variables were recorded at the initiation of the cycle, while categorical variables were classified according to standard clinical classifications. The definitions of all variables followed the glossary of the International Committee for Monitoring Assisted Reproductive Technology (ICMART) (19).

### Data preprocessing and feature engineering

Continuous variables were standardized using z-score normalization (mean=0, SD=1) to ensure comparable scales across features. Categorical variables were label-encoded into numerical format. The dataset was partitioned into training (70%) and test (30%) sets using stratified random sampling to maintain outcome distribution.

### Feature selection

We employed XGBoost-based feature importance ranking to identify the most predictive variables (20). To avoid conditioning the analytic cohort on variables that were not ultimately retained, feature selection was performed on all screened cycles using XGBoost’s native handling of missing values, so that no record required deletion at this stage. To prevent information leakage, the ranking model was fitted on the training partition only, with 100 estimators, maximum depth of 6, and learning rate of 0.1. Features were ranked by gain importance, and the top eight features were selected for subsequent modeling to balance predictive performance with model parsimony and clinical feasibility. All eight selected predictors were recorded in every screened cycle; consequently the analytic cohort comprised all 7,773 cycles and no cycle was excluded because of missing predictor data.

### Model selection and training

We assessed the performance of six distinct machine learning algorithms: (1) logistic regression with L1/L2 regularization (liblinear solver, maximum 1000 iterations) (21); (2) decision tree classifier (maximum depth=10, minimum samples split=20, minimum samples leaf=10) (22); (3) AdaBoost classifier (100 estimators, learning rate=0.8) (23); (4) bagging classifier (100 estimators, 80% sample and feature bootstrapping) (24); (5) gradient boosting classifier (default parameters) (20); and (6) Gaussian naive Bayes (25).

These six algorithms were selected to span a graded spectrum from fully interpretable linear models to flexible non-linear ensembles, consistent with reporting guidance to benchmark novel machine-learning approaches against simpler, well-established alternatives rather than evaluating them in isolation (30). Each has an extensively validated, standard implementation in the general prediction-modelling literature; more recent gradient-boosting variants (e.g., LightGBM, CatBoost) and deep tabular-learning architectures were not benchmarked head-to-head in this study and are noted as a direction for future work (see Limitations).

To ensure internal validity and mitigate the risk of overfitting, we employed stratified 10-fold cross-validation on the training set. For each iteration, the model was trained on nine of the folds and tested on the remaining fold, with all ten combinations being evaluated. The performance metrics were then averaged across the folds to yield stable and reliable estimates with minimized variance. This procedure was repeated for each of the six algorithms, facilitating a rigorous and unbiased comparison of their performance.

### Model evaluation metrics

The primary evaluation metric was the area under the receiver operating characteristic curve (AUC), computed using the trapezoidal rule. Secondary metrics included accuracy, sensitivity (recall), specificity, positive predictive value (PPV), negative predictive value (NPV), F1-score, precision, and the Matthews correlation coefficient (MCC) (26).

For the final test set assessment, 95% confidence intervals for AUC were derived using 2000 bootstrap resamples, with bias-corrected and accelerated adjustment applied. Bootstrap resampling maintained stratification to ensure consistent outcome prevalence. The model with the highest AUC and satisfactory performance across secondary metrics was selected as the optimal predictor.

### Model validation and clinical utility evaluation

#### Calibration assessment

Calibration curves were generated to assess the concordance between predicted probabilities and actual outcomes (27). The predicted probabilities were divided into deciles based on quantiles, and the observed proportion of positive outcomes for each decile was plotted against the mean predicted probability. Ideal calibration was represented by the 45° reference line, indicating a perfect match between predictions and observed frequencies.

#### Decision curve analysis

To evaluate the clinical utility of the model, decision curve analysis (DCA) was employed, which calculates the net benefit across a range of threshold probabilities (from 0.01 to 0.99) (28). The net benefit was defined as follows:


Net Benefit = (True Positives/N) − (False Positives/N) × [Threshold/(1 − Threshold)],

where N denotes the sample size. The model’s net benefit was compared against the “treat all” and “treat none” strategies, thereby identifying the threshold probability ranges that maximize clinical utility and offer actionable decision support.

### SHAP interpretability

To enhance the clinical interpretability of the model, we employed SHapley Additive exPlanations (SHAP) for the top-performing model (16, 29). SHAP values were computed for all observations in the test set, providing a detailed assessment of each feature’s contribution to individual predictions. The following visualizations were generated to facilitate understanding:

Summary Plots: Beeswarm plots were created to illustrate the global feature importance and the directional effects of each feature, highlighting how different predictors influence the overall model output.
Dependence Plots: Scatter plots were produced to explore the relationships between the top five predictors and the predicted outcomes, providing insight into how these key features interact with the target variable.

Feature importance was quantified by calculating the mean absolute SHAP value, which was then exported for clinical interpretation. This approach not only supports transparency but also provides actionable insights into the model’s decision-making process, enabling clinicians to better understand the underlying drivers of predictions and improve decision-making in practice.

### Sensitivity analyses

#### Missing data handling strategy

Because the eight predictors of the primary model were complete in all cycles, the primary analysis required no imputation. To verify that this was not an artefact of the selected feature set, we repeated the analysis using the twelve highest-ranked predictors, among which variables ranked ninth and beyond contain missing values, and compared complete-case analysis (listwise deletion) with multivariate imputation by chained equations (scikit-learn IterativeImputer, 10 iterations), fitted on the training partition and applied to the test partition.

#### Feature selection stability

To evaluate the robustness of feature selection, we varied the number of features retained (top 3, 5, 8, 10, and 12 features) and systematically assessed model performance across these different configurations. This analysis enabled us to determine the optimal feature set size that balanced predictive accuracy with clinical relevance, ensuring that the model remained both effective and interpretable in real-world clinical settings.

### Statistical analysis

All analyses were performed using Python 3.10 (Python Software Foundation). Machine learning models were implemented using scikit-learn and XGBoost. Model interpretability was assessed using SHAP. Data manipulation and preprocessing were conducted with NumPy and Pandas, while data visualization utilized Matplotlib and Seaborn.

This study was reported in accordance with the TRIPOD+AI (Transparent Reporting of a multivariable prediction model for Individual Prognosis Or Diagnosis, extended for artificial intelligence) statement (30) for prediction-model development and validation. Risk of bias was self-assessed using the Prediction model Risk Of Bias ASsessment Tool (PROBAST) (31) across the participants, predictors, outcome, and analysis domains; the study was judged to be at low-to-moderate risk of bias, with the principal residual concern being the single-center design, partially mitigated by temporal validation.

Baseline characteristics were summarized and stratified by outcome status. The Shapiro-Wilk test was applied to assess normality for continuous variables. Normally distributed variables are presented as mean ± standard deviation (SD) and compared using independent samples t-tests, with Welch’s correction applied when Levene’s test indicated heterogeneity of variance (p<0.05). Non-normally distributed variables are reported as median (interquartile range [IQR]) and compared using the Mann-Whitney U test. Categorical variables are expressed as frequency (percentage) and analyzed using Pearson’s χ² test or Fisher’s exact test when expected cell frequencies were<5.

All statistical tests were two-sided, with statistical significance defined as p<0.05. All statistical analyses were conducted using Python’s SciPy and Statsmodels libraries.

### Temporal validation

To emulate prospective clinical deployment, we performed temporal (out-of-time) validation in addition to random splitting. The model was trained exclusively on cycles performed between January 2019 and December 2022 (n=6,166) and evaluated on an independent, chronologically later cohort of cycles performed between January 2023 and June 2024 (n=1,607). Identical feature-selection and training procedures were applied. Because all eight model predictors were complete in every cycle, the temporal analysis used the same 7,773 cycles as the primary analysis, partitioned chronologically rather than at random; no imputation was required. Because temporal validation assesses model transportability to future patients rather than to a random subsample of the same period, it provides a more stringent and clinically realistic estimate of generalizability. This accords with methodological guidance that external and temporal validation provide a more rigorous test of a prediction model’s transportability than internal random-split validation alone (32).

### Benchmark comparison with an established prognostic model and an information-matched model

To contextualize performance against the current clinical standard, we constructed a reference logistic-regression model approximating the McLernon pre-treatment framework, the best-performing published cumulative live-birth prediction model. This benchmark used the four predictors most closely corresponding to the McLernon variable set available in our data: female age, treatment cycle number (a proxy for the number of previous complete cycles), infertility type, and parity (previous live birth). Because this benchmark differs from the proposed model both in algorithm and in the information available to it, a comparison between the two cannot attribute any difference to either factor alone. We therefore added a third, information-matched model: a gradient-boosting classifier restricted to pre-treatment variables only. Contrasting this model with the logistic benchmark isolates the contribution of the learning algorithm, whereas contrasting it with the Top-8 model isolates the contribution of embryological information. All three models were compared on the same test cases using DeLong’s test for correlated ROC curves, together with the continuous Net Reclassification Improvement (NRI) and Integrated Discrimination Improvement (IDI), each with percentile 95% confidence intervals from 2,000 bootstrap resamples.

### Subgroup calibration analysis

Because prognostic accuracy is most consequential in older patients, we assessed model performance within clinically defined female-age strata (<35, 35–37, 38–40, and >40 years). For each stratum we computed the AUC with bootstrap 95% confidence intervals, the Brier score, and calibration curves comparing predicted probabilities with observed cumulative live-birth rates.

### Dynamic sequential prediction

To quantify how prognostic information accrues across the ART treatment pathway, we developed a dynamic (sequential) prediction framework in which the cumulative live-birth model was reconstructed at three clinically meaningful decision points using only the variables available at each stage. Stage 1 (pre-treatment) used demographic, ovarian-reserve, and reproductive-history variables available before treatment initiation. Stage 2 (post-retrieval) additionally incorporated endocrine markers and ovarian-response parameters (follicle counts and oocytes retrieved). Stage 3 (post-transfer) further added embryological and transfer parameters, including embryo quality, developmental stage, number of embryos transferred, and endometrial characteristics. Each stage-specific model was trained and evaluated using the identical partition, cross-validation, and gradient-boosting configuration as the primary analysis, enabling direct comparison of discriminatory gain across decision points.

## Results

### Study population and baseline characteristics

All 7,773 screened oocyte retrieval cycles were included in the analytic cohort; because every one of the eight model predictors was recorded in all cycles, no cycle was excluded for missing predictor data. Using stratified random sampling, cycles were allocated to a training set (n=5,441; 70%) and a test set (n=2,332; 30%). The cumulative live birth rate was 34.6% in the training set (1,884/5,441) and 34.6% in the test set (808/2,332), confirming successful stratification of the primary outcome. Baseline characteristics were highly comparable between sets (**Table 1**). The median female age was 34 years in both groups (IQR, 30–38). The total follicle count (≥14 mm) was 8 (IQR, 4–11), and the number of oocytes retrieved was 10 (IQR, 5–16) in the training set and 11 (IQR, 6–16) in the test set.

**Table 1:** Baseline characteristics of the training and test sets (n = 7,773 oocyte retrieval cycles).

Characteristic	Training set (n=5,441)	Test set (n=2,332)	P value
No. of cycles	5,441	2,332	
Cumulative live birth, n (%)	1,884 (34.6)	808 (34.6)	1.000
Female age (median, IQR)	34.00 (30.00, 38.00)	34.00 (30.00, 38.00)	0.488
Male age (median, IQR)	35.00 (32.00, 39.00)	35.00 (32.00, 39.00)	0.982
BMI (median, IQR)	21.78 (20.00, 24.00)	22.03 (20.20, 24.03)	0.016
AMH (median, IQR)	2.17 (1.32, 3.63)	2.17 (1.32, 3.77)	0.259
Duration of infertility (median, IQR)	4.00 (2.00, 6.17)	4.00 (2.00, 6.25)	0.955
Gravidity (median, IQR)	1.00 (0.00, 2.00)	1.00 (0.00, 2.00)	0.680
Parity (median, IQR)	0.00 (0.00, 1.00)	0.00 (0.00, 1.00)	0.386
Abortions (median, IQR)	0.00 (0.00, 1.00)	0.00 (0.00, 1.00)	0.837
LH (median, IQR)	2.05 (1.40, 3.20)	2.03 (1.39, 3.16)	0.678
Estradiol (median, IQR)	2222.50 (1302.00, 3700.25)	2265.00 (1357.00, 3686.00)	0.611
Progesterone (median, IQR)	0.56 (0.37, 0.82)	0.56 (0.37, 0.82)	0.976
Total follicles >=14mm (median, IQR)	8.00 (4.00, 11.00)	8.00 (4.00, 11.00)	0.871
Oocytes retrieved (median, IQR)	10.00 (5.00, 16.00)	11.00 (6.00, 16.00)	0.413
Endometrial thickness (median, IQR)	10.00 (8.00, 11.00)	10.00 (8.90, 11.00)	0.584
Treatment cycle, n (%)			0.994
1	3,610 (66.3)	1,548 (66.4)	
2	1,212 (22.3)	514 (22.0)	
3	406 (7.5)	171 (7.3)	
4	127 (2.3)	57 (2.4)	
5	50 (0.9)	25 (1.1)	
6	24 (0.4)	10 (0.4)	
7	8 (0.1)	5 (0.2)	
8	4 (0.1)	2 (0.1)	
Transfer type, n (%)			0.537
0	2,906 (53.4)	1,227 (52.6)	
1	2,535 (46.6)	1,105 (47.4)	
Protocol, n (%)			0.652
0	665 (12.2)	283 (12.1)	
1	75 (1.4)	33 (1.4)	
2	1,191 (21.9)	515 (22.1)	
3	59 (1.1)	18 (0.8)	
4	56 (1.0)	30 (1.3)	
5	88 (1.6)	26 (1.1)	
6	994 (18.3)	433 (18.6)	
7	115 (2.1)	43 (1.8)	
8	1,594 (29.3)	676 (29.0)	
9	604 (11.1)	275 (11.8)	
Embryos transferred, n (%)			0.267
1	1,704 (31.3)	700 (30.0)	
2	3,737 (68.7)	1,632 (70.0)	
Embryo quality (grade), n (%)			0.649
1	462 (8.5)	181 (7.8)	
2	842 (15.5)	364 (15.6)	
3	1,242 (22.8)	519 (22.3)	
4	2,050 (37.7)	881 (37.8)	
5	845 (15.5)	387 (16.6)	
Embryo stage, n (%)			0.677
1	4,343 (79.8)	1,875 (80.4)	
2	682 (12.5)	292 (12.5)	
3	416 (7.6)	165 (7.1)	
Infertility type, n (%)			0.209
0	1,963 (36.1)	877 (37.6)	
1	3,478 (63.9)	1,455 (62.4)	
Endometrial pattern, n (%)			0.390
1.0	45 (0.8)	16 (0.7)	
2.0	25 (0.5)	14 (0.6)	
3.0	83 (1.5)	24 (1.0)	
4.0	800 (14.7)	340 (14.6)	
5.0	4,421 (81.3)	1,914 (82.1)	

Continuous variables are presented as mean ± SD (normally distributed, compared by independent-samples t test) or median (interquartile range) (non-normally distributed, compared by Mann-Whitney U test); normality was assessed by the Shapiro-Wilk test. Categorical variables are presented as n (%) and compared by Pearson chi-square test. Embryo quality is graded 1 (lowest) to 5 (highest). All 7,773 screened cycles were analysed; no cycle was excluded for missing data because all eight model predictors were complete.

Regarding treatment characteristics, most cycles were first treatment cycles (66.3% training; 66.4% test), with decreasing proportions thereafter. Fresh embryo transfer occurred in 53.4% of training cycles and 52.6% of test cycles; the remainder involved frozen–thawed transfers. Protocol distributions were similarly balanced between the two sets, and no baseline characteristic differed materially between them (**Table 1**).

Embryo quality distributions were likewise aligned: Grade 4 embryos constituted the largest share (37.7% training vs 37.8% test), followed by Grade 3 (22.8% vs 22.3%) and Grade 2 (15.5% vs 15.6%). As per the grading rubric (Grade 5 highest quality; Grade 1 lowest), high-quality Grade 5 embryos accounted for 15.5% versus 16.6% and low-quality Grade 1 embryos for 8.5% versus 7.8%. Embryo developmental stage was likewise comparable, with cleavage-stage transfers predominating (79.8% of training cycles).

### Feature selection

XGBoost-based feature importance ranking identified eight key predictive variables from the initial feature set (**Figure 2**). Embryo quality emerged as the single most important predictor with a relative gain of 0.1404, substantially exceeding all other variables. The remaining seven features demonstrated progressively lower gain: treatment cycle number (0.0864), female age (0.0805), transfer type (fresh vs frozen–thawed, 0.0709), number of embryos transferred (0.0444), treatment protocol (0.0386), embryo developmental stage (0.0293), and parity (0.0240). Together these eight features accounted for 51.5% of the total gain, with embryo quality alone contributing approximately 14%. All eight were complete in every screened cycle and were selected for subsequent model development.

**Figure 2:**
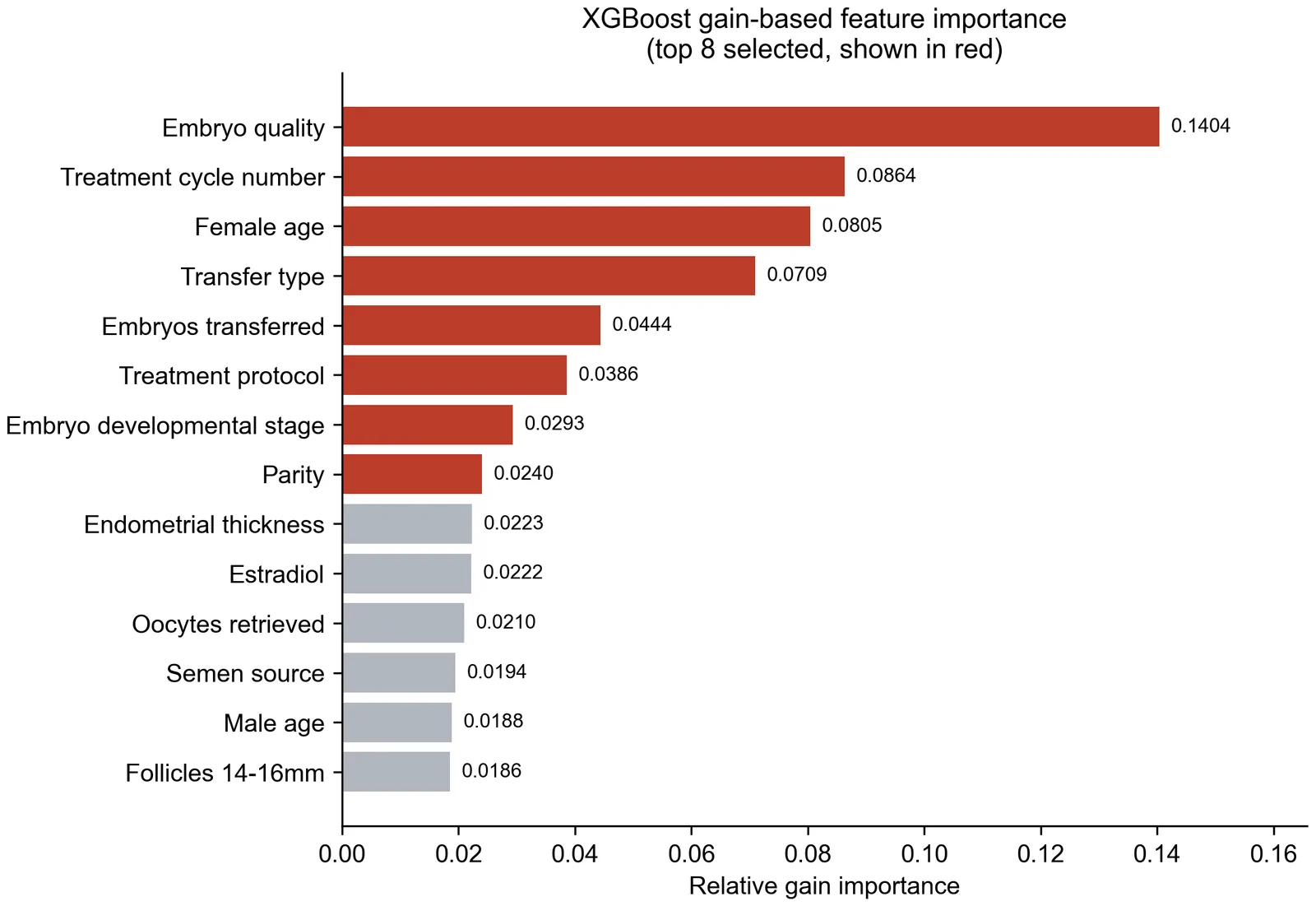
Feature importance ranking and selection using XGBoost. Gain-based feature importance (training partition). The eight selected predictors are shown in red; the next six ranked variables are shown in grey for reference.

### Model performance: cross-validation results

Six machine learning algorithms were systematically evaluated using stratified 10-fold cross-validation on the training set (n=5,441) (**Table 2**, **Figure 3**). Gradient Boosting demonstrated superior discriminative performance with a mean AUC of 0.786 (95% CI: 0.737–0.835), outperforming AdaBoost (0.774, 95% CI: 0.720–0.829), Logistic Regression (0.760, 95% CI: 0.704–0.816), Decision Tree (0.758, 95% CI: 0.704–0.812), Bagging Classifier (0.758, 95% CI: 0.711–0.805), and Gaussian Naive Bayes (0.745, 95% CI: 0.694–0.796) (**Figure 3A**). Confidence intervals derived from between-fold variability overlapped across algorithms, so the ranking should be read as a consistent ordering of point estimates rather than as evidence of statistically distinct performance (**Figure 3B**).

**Table 2:** Discrimination and calibration of six machine-learning algorithms under stratified 10-fold cross-validation in the training set (n = 5,441).

Model	Accuracy	AUC	95% CI	Sensitivity	Specificity	PPV	NPV	F1-Score	MCC
Gradient Boosting	0.733	0.786	[0.737-0.835]	0.583	0.812	0.623	0.786	0.602	0.402
AdaBoost	0.719	0.774	[0.720-0.829]	0.505	0.833	0.616	0.761	0.554	0.357
Logistic Regression	0.707	0.760	[0.704-0.816]	0.456	0.840	0.603	0.745	0.518	0.321
Decision Tree	0.706	0.758	[0.704-0.812]	0.589	0.768	0.573	0.779	0.580	0.355
Bagging Classifier	0.717	0.758	[0.711-0.805]	0.559	0.800	0.597	0.775	0.577	0.365
Gaussian Naive Bayes	0.687	0.745	[0.694-0.796]	0.643	0.711	0.542	0.790	0.587	0.343

Values are means across 10 stratified folds (training set n=5,441). The 95% CI for AUC is derived from between-fold variability. AUC, area under the receiver operating characteristic curve; CI, confidence interval; PPV, positive predictive value; NPV, negative predictive value; MCC, Matthews correlation coefficient.

**Figure 3:**
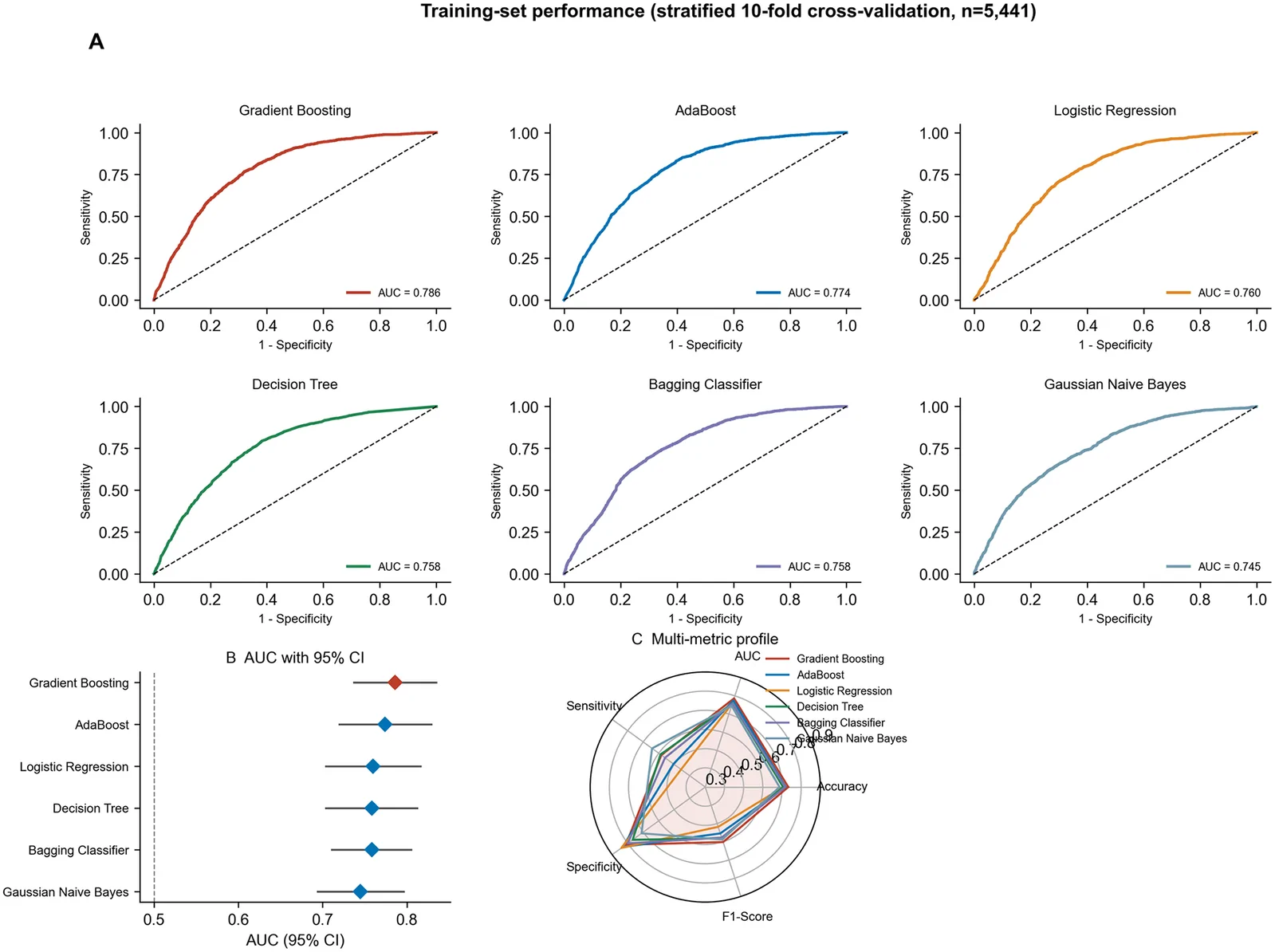
Training set cross-validation performance. **(A)** ROC curves for six models under stratified 10-fold cross-validation (n=5,441; pooled out-of-fold predictions). **(B)** Forest plot of AUC with 95% CIs from between-fold variability. **(C)** Multi-metric radar chart across five evaluation criteria.

The Gradient Boosting model achieved cross-validation accuracy of 0.733, with sensitivity of 0.583 and specificity of 0.812, demonstrating well-balanced classification performance. The positive and negative predictive values were 0.623 and 0.786, respectively. The F1-score was 0.602 and the Matthews correlation coefficient 0.402, the highest among the six algorithms.

Multi-metric radar chart analysis revealed distinct performance profiles across models (**Figure 3C**). Gaussian Naive Bayes exhibited the highest sensitivity (0.643) but at the cost of reduced specificity (0.711), resulting in a trade-off between true positive and false positive rates. In contrast, Gradient Boosting achieved the most balanced profile, combining the highest AUC with competitive values across all secondary metrics.

### Testing set performance

All six models were retrained on the complete training set and evaluated on the independent test set (n=2,332) (**Table 3**, **Figure 4**). Gradient Boosting maintained superior discriminative performance with an AUC of 0.762 (95% CI: 0.743–0.781), followed by AdaBoost (0.748, 95% CI: 0.728–0.769), Decision Tree (0.744, 95% CI: 0.723–0.763), Bagging Classifier (0.739, 95% CI: 0.719–0.758), Logistic Regression (0.737, 95% CI: 0.715–0.757), and Gaussian Naive Bayes (0.719, 95% CI: 0.697–0.740) (**Figure 4A**). Forest plot analysis confirmed that Gradient Boosting achieved the highest point estimate with relatively narrow confidence intervals, though there was substantial overlap with other ensemble methods, indicating comparable discriminative ability (**Figure 4B**).

**Table 3:** Performance of the six algorithms in the independent random-split test set (n = 2,332).

Model	Accuracy	AUC	95% CI	Sensitivity	Specificity	PPV	NPV	F1-Score	MCC	Brier score
Gradient Boosting	0.710	0.762	[0.743-0.781]	0.556	0.791	0.585	0.771	0.570	0.351	0.185
AdaBoost	0.696	0.748	[0.728-0.769]	0.475	0.814	0.575	0.745	0.520	0.304	0.206
Decision Tree	0.695	0.744	[0.723-0.763]	0.585	0.753	0.556	0.774	0.571	0.334	0.194
Bagging Classifier	0.695	0.739	[0.719-0.758]	0.551	0.771	0.560	0.764	0.556	0.323	0.193
Logistic Regression	0.689	0.737	[0.715-0.757]	0.444	0.818	0.564	0.735	0.497	0.281	0.193
Gaussian Naive Bayes	0.659	0.719	[0.697-0.740]	0.603	0.688	0.506	0.766	0.550	0.281	0.233

The 95% CI for AUC is derived from 2,000 bootstrap resamples. Classification metrics use a probability threshold of 0.5. Lower Brier scores indicate better probability accuracy. Abbreviations as in **Table 2**.

**Figure 4:**
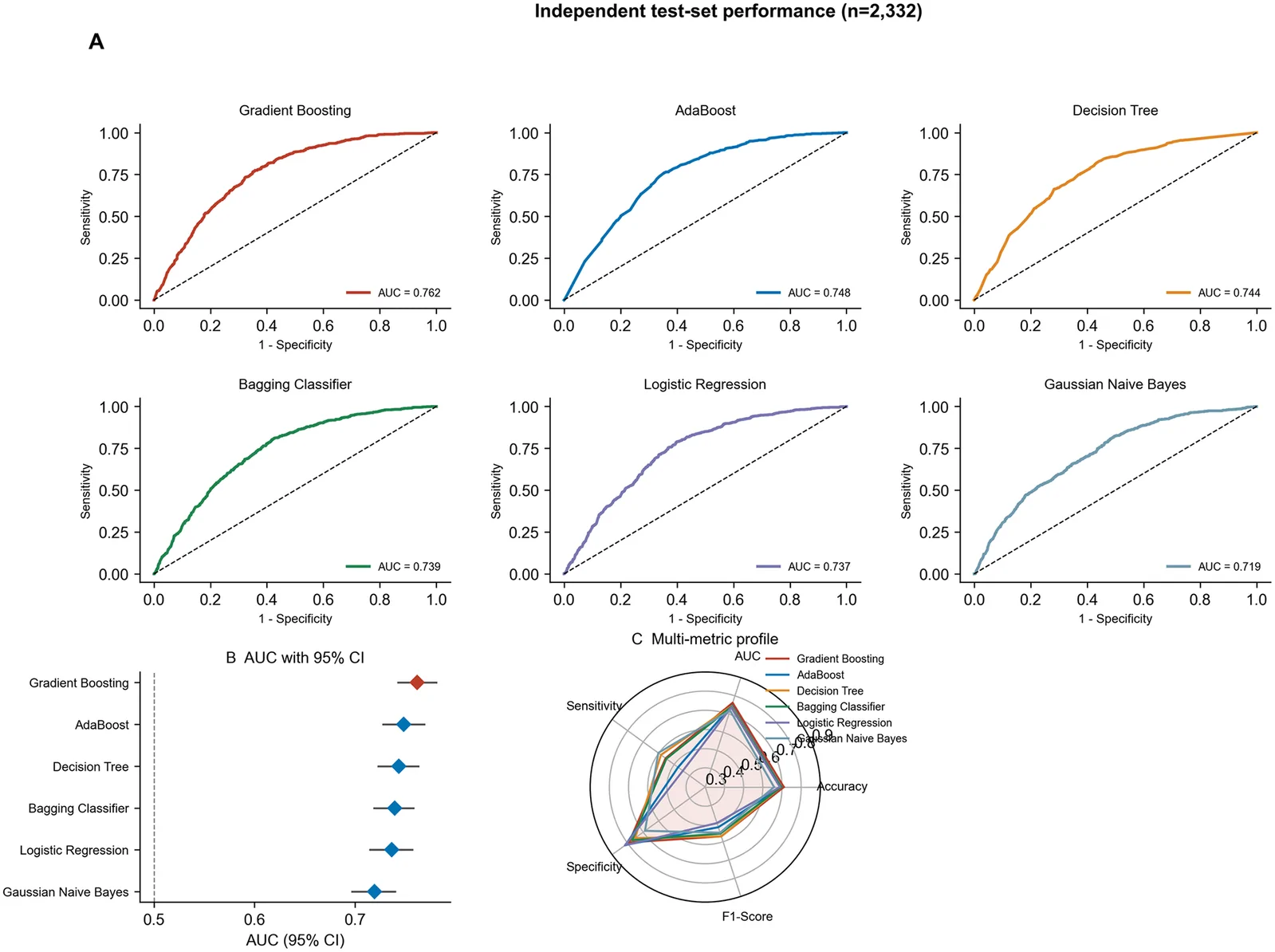
Independent test set performance. **(A)** ROC curves for six models on the independent test set (n=2,332). **(B)** Forest plot of AUC with bootstrap 95% CIs. **(C)** Multi-metric radar chart across five evaluation criteria.

The Gradient Boosting model achieved test set accuracy of 0.710, with sensitivity of 0.556 and specificity of 0.791, maintaining well-balanced performance between true positive and false positive rates. The positive and negative predictive values were 0.585 and 0.771, respectively. The F1-score was 0.570 and the Matthews correlation coefficient 0.351.

Multi-metric radar chart analysis on the test set revealed performance patterns consistent with cross-validation findings (**Figure 4C**). Gaussian Naive Bayes exhibited the highest sensitivity (0.603) but with the lowest specificity (0.688), maintaining its characteristic trade-off profile. Gradient Boosting retained the highest AUC, accuracy, and Matthews correlation coefficient, indicating the most favourable overall balance among the six algorithms.

Compared with the logistic regression model fitted to the same eight predictors, Gradient Boosting achieved an absolute AUC improvement of 0.025 (0.762 vs 0.737), accompanied by higher accuracy (0.710 vs 0.689) and sensitivity (0.556 vs 0.444) at slightly lower specificity (0.791 vs 0.818). As shown in the benchmark comparison below, however, a difference of this magnitude was not reproduced when the two model classes were given an identical, purely pre-treatment variable set, indicating that algorithm choice contributes far less than the information available to the model.

### Model calibration and clinical utility

Calibration assessment evaluated the concordance between predicted probabilities and observed outcomes across all models (**Figure 5A**). The calibration curves revealed variable performance patterns, with most models demonstrating reasonable agreement with the 45° perfect calibration line across the mid-range probabilities (0.3-0.7). Gradient Boosting and Logistic Regression exhibited the closest adherence to ideal calibration, with predicted probabilities closely matching observed event frequencies across most probability bins. Notably, AdaBoost displayed substantial calibration deviation in the mid-probability range (0.4-0.6), with observed frequencies markedly higher than predicted probabilities, indicating systematic underestimation of risk in this critical decision-making range. Gaussian Naive Bayes showed slight overestimation at lower predicted probabilities and underestimation at higher probabilities, reflecting characteristic calibration challenges of probabilistic classifiers.

**Figure 5:**
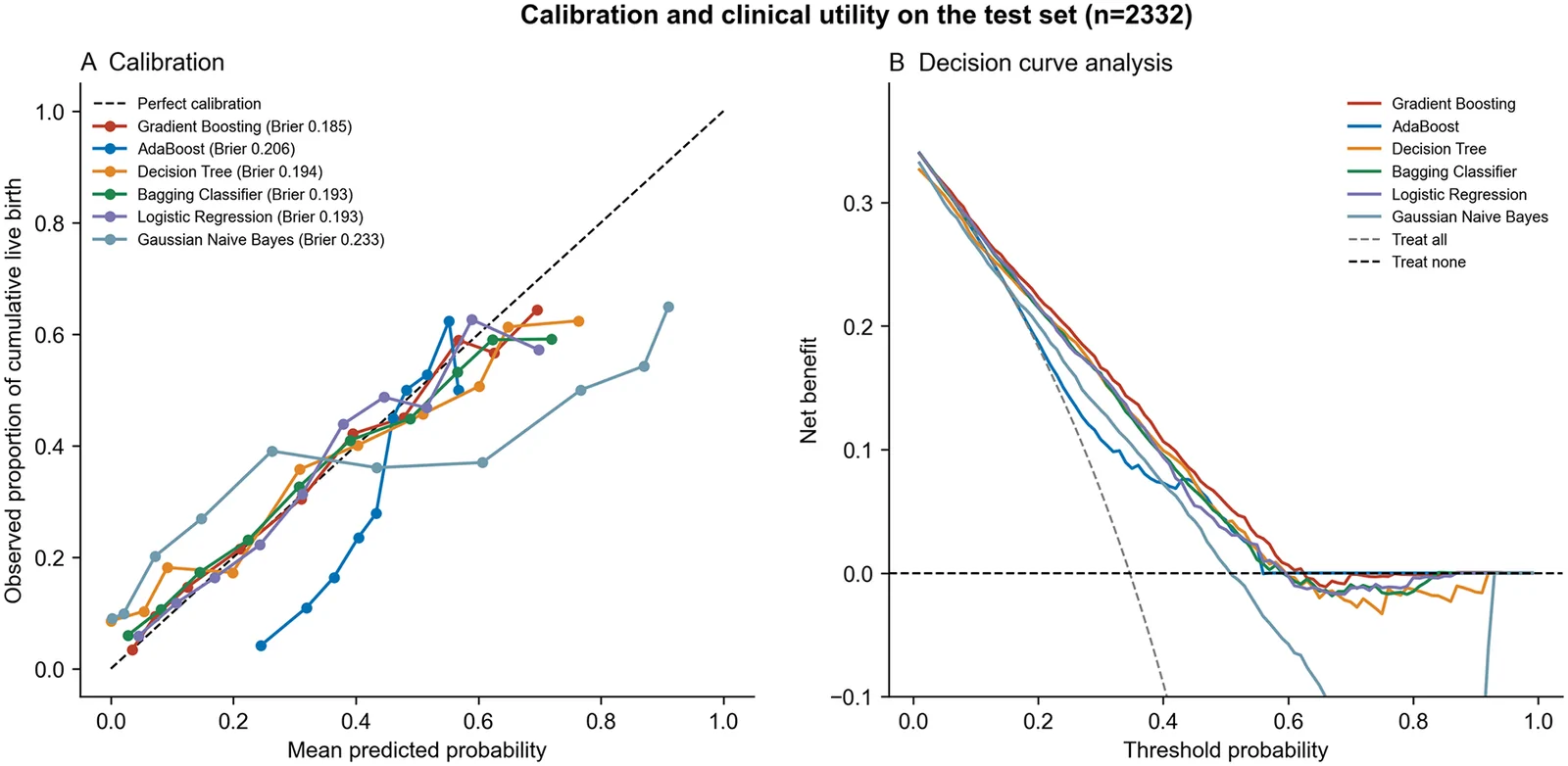
Model calibration and clinical utility assessment. **(A)** Calibration curves (quantile-based deciles) for all six models on the test set (n=2,332); the dashed diagonal indicates perfect calibration. **(B)** Decision curve analysis of net benefit across threshold probabilities, compared with the “treat all” and “treat none” strategies.

The Brier score, which quantifies both calibration and discrimination (lower values indicate better performance), confirmed these observations (**Table 3**). Gradient Boosting achieved the lowest Brier score (0.185), indicating superior probability accuracy compared to Bagging Classifier (0.193), Logistic Regression (0.192), Decision Tree (0.204), Gaussian Naive Bayes (0.217), and AdaBoost (0.247). The substantially higher Brier score for AdaBoost (0.247) aligned with its poor calibration curve performance, suggesting that despite reasonable discriminative ability (AUC 0.748), its probability estimates were less reliable for clinical decision-making. The comparable Brier scores between Gradient Boosting (0.185) and Logistic Regression (0.192) indicate that both models provide similarly reliable probability estimates, though Gradient Boosting maintained a slight advantage.

Decision curve analysis assessed clinical utility by quantifying net benefit across threshold probabilities ranging from 0.01 to 0.99 (**Figure 5B**). All models demonstrated positive net benefit compared with the “treat none” strategy across threshold probabilities from 0.01 to approximately 0.94, and exceeded the “treat all” strategy across essentially the entire range examined. The models converged with nearly identical net benefit curves, overlapping extensively across the clinically relevant threshold range (0.2–0.5), suggesting comparable clinical value despite differences in AUC. Maximum incremental benefit relative to the “treat all” strategy was observed at thresholds between 0.30 and 0.40.

### Model interpretability: SHAP analysis

To enhance clinical interpretability and transparency, we conducted SHAP (SHapley Additive exPlanations) analysis for the Gradient Boosting model on the test set (n=2,332) (**Figure 6**). SHAP values quantify each feature’s contribution to individual predictions, enabling insight into the model’s decision process.

**Figure 6:**
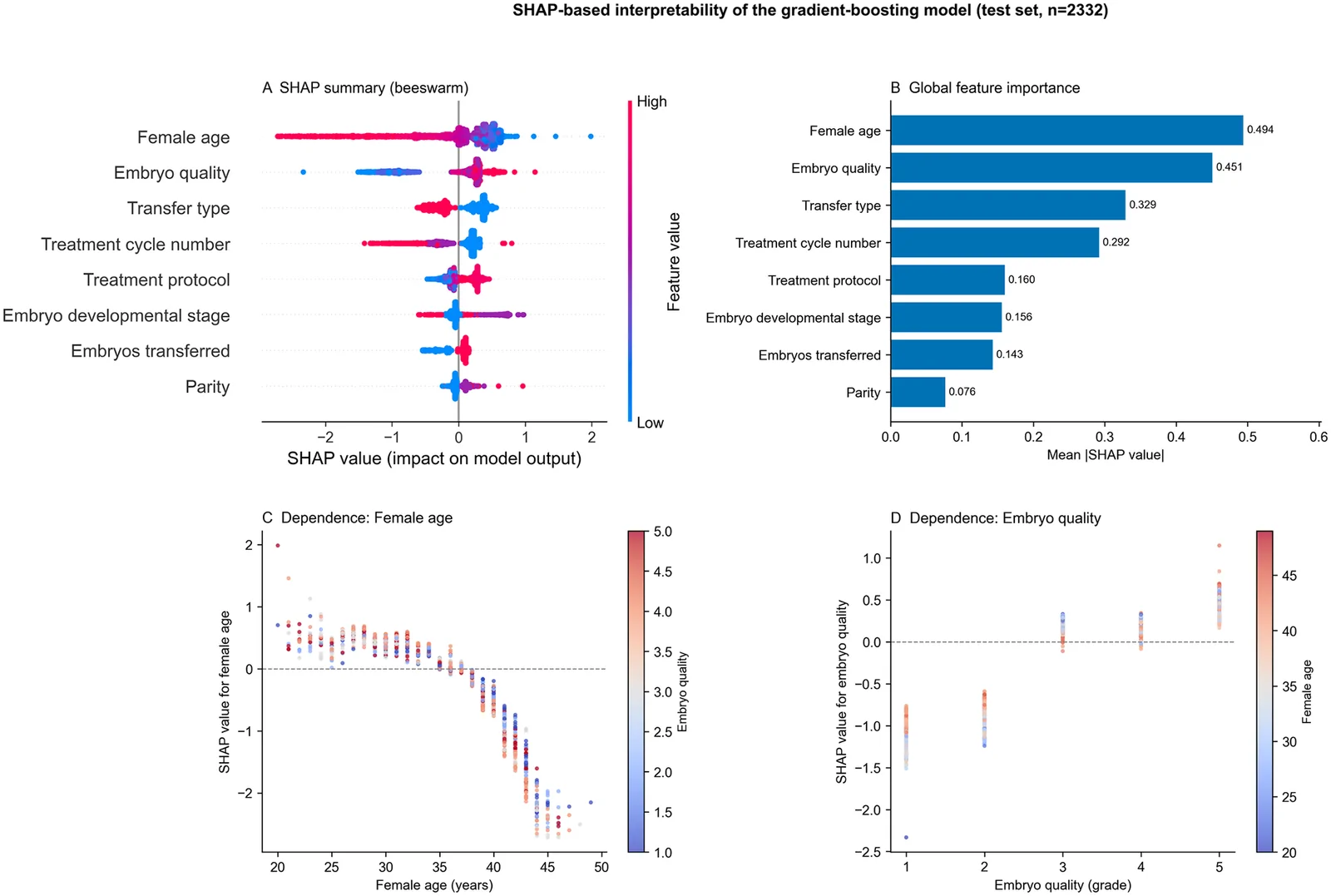
SHAP-based interpretability of the gradient boosting model (test set, n=2,332). **(A)** Summary (beeswarm) plot of global feature importance and directional effects; colour encodes feature value. **(B)** Mean absolute SHAP value by feature. **(C)** Dependence plot for female age (colour encodes embryo quality). **(D)** Dependence plot for embryo quality on the clinical 1–5 grading scale (colour encodes female age).

### Global feature importance

Analysis of mean absolute SHAP values identified female age as the most influential predictor (0.494), followed by embryo quality (0.451), transfer type (0.329), and treatment cycle number (0.292) (**Figures 6A, B**). These four features account for the majority of predictive power. The remaining features—treatment protocol (0.160), embryo developmental stage (0.156), number of embryos transferred (0.143), and parity (0.076)—provide secondary, fine-grained refinement. Notably, the SHAP-based ranking differs from XGBoost gain importance: female age surpasses embryo quality, suggesting that while embryo quality offers strong discriminative information at the population level, age exerts greater influence on individual-level variability.

### Age-related impact patterns

The SHAP summary plot shows that higher female age contributes predominantly negative SHAP values, with individual values spanning +1.99 to −2.72 across the cohort (**Figure 6A**). The dependence plot, presented on the original age scale, demonstrates a plateau through the early thirties followed by an accelerating decline (**Figure 6C**). Mean SHAP contribution remained positive up to age 37 (+0.02 at 37 years), crossed zero between 37 and 38 years (−0.16 at 38 years), and fell steeply thereafter (−0.51 at 40 years; −1.63 at 43 years; −2.33 at 45 years). This pattern confirms the well-established inverse relationship between maternal age and live-birth probability, with an inflection in the 38–40 year range and an accelerated drop beyond it. The consistent direction and magnitude across individuals underscore age as the principal non-modifiable prognostic factor.

### Embryo quality impact patterns

Embryo quality exhibits a clear stepwise gradient in SHAP contributions: Grade 1 embryos yield strongly negative values (mean −1.16) and Grade 2 remain negative (−0.94), whereas Grades 3 through 5 contribute positively (+0.24, +0.25, and +0.42, respectively) (**Figures 6A, D**). The dependence plot, presented on the clinical 1–5 grading scale, reveals discrete rather than continuous effects, with the largest single increment occurring between Grade 2 and Grade 3.

The remaining features (transfer type, treatment cycle number, treatment protocol, embryo developmental stage, and parity) show smaller SHAP magnitudes clustered near zero, indicating secondary yet meaningful roles in refining predictions. Overall, SHAP analysis shows that the Gradient Boosting model centers on established clinical determinants of cumulative live birth, supporting the biological plausibility of its predictions.

### Sensitivity analyses

Two sensitivity analyses were conducted to assess the robustness of the Gradient Boosting model’s performance under different analytical conditions.

### Missing data handling

Because all eight predictors of the primary model were complete, the primary analysis involved no missing data and required no imputation. To confirm that this reflected the properties of the selected predictors rather than a favourable subsample, we repeated the analysis on the twelve highest-ranked predictors, four of which contain missing values (**Figure 7A**). Complete-case analysis retained 2,267 test cycles and achieved an AUC of 0.761, whereas multivariate imputation by chained equations preserved all 2,332 test cycles and yielded an AUC of 0.763. The ROC curves demonstrated almost complete overlap, with a difference in AUC of 0.002 and no statistically meaningful difference in discriminative performance, indicating that missing-data handling did not materially affect model behaviour.

**Figure 7:**
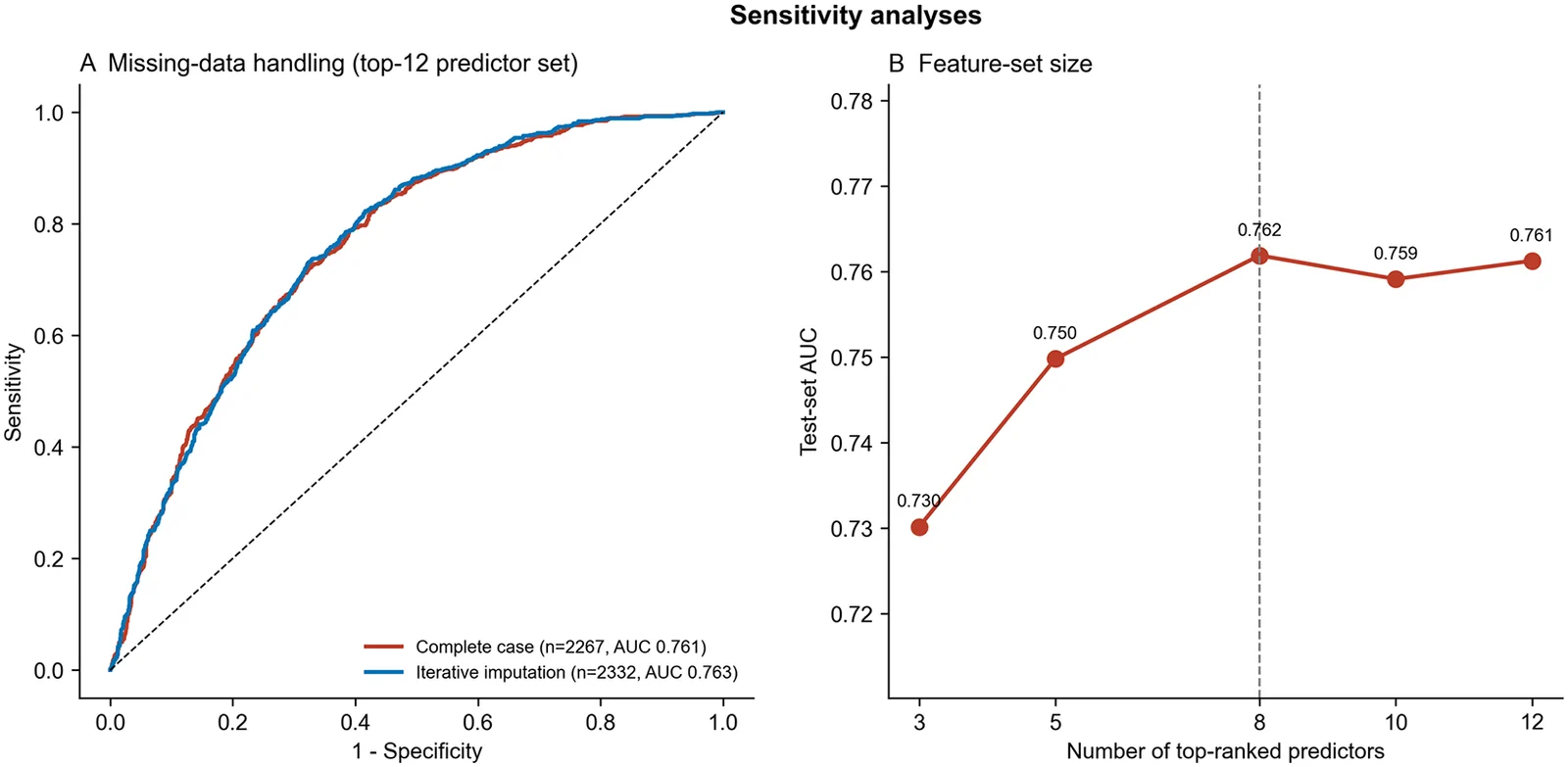
Sensitivity analyses. **(A)** ROC curves comparing complete-case analysis with multivariate imputation for the twelve-predictor model. **(B)** Test-set AUC as a function of the number of top-ranked predictors (3, 5, 8, 10, 12); the dashed line marks the selected 8-feature model.

### Feature selection sensitivity

The impact of feature set size on model performance was systematically evaluated using top-ranked features from XGBoost importance analysis (**Figure 7B**, **Table 4**). A minimal feature set using only the top 3 predictors achieved AUC of 0.730 and accuracy of 0.693. Expanding to 5 features improved performance (AUC 0.750, accuracy 0.700), and the primary 8-feature model achieved the highest AUC of the series (0.762) with accuracy 0.710.

**Table 4:** Sensitivity analysis of the number of retained predictors (test set, n = 2,332).

No. of features	Test cycles analysed	AUC	Accuracy	Sensitivity	Specificity	PPV	NPV	F1-Score
3	2332	0.730	0.693	0.558	0.764	0.557	0.765	0.557
5	2332	0.750	0.700	0.557	0.776	0.569	0.768	0.563
8	2332	0.762	0.710	0.556	0.791	0.585	0.771	0.570
10	2271	0.759	0.711	0.541	0.801	0.589	0.768	0.564
12	2267	0.761	0.712	0.538	0.803	0.591	0.767	0.563

Features were added in order of XGBoost gain importance. The number of test cycles varies across feature sets because predictors ranked 9th and beyond contain missing values, whereas the eight predictors of the primary model are complete in all cycles. Abbreviations as in **Table 2**.

Further expansion conferred no additional benefit: models using the top 10 and top 12 predictors achieved AUCs of 0.759 and 0.761 respectively, marginally below the 8-feature model, with sensitivity declining from 0.556 to 0.538 as specificity rose. Because predictors ranked ninth and beyond contain missing values, these larger models were also fitted to slightly fewer cycles (2,271 and 2,267 test cycles, respectively). Performance therefore peaked at eight features, supporting the decision to prioritize model parsimony and complete data capture over further expansion of the predictor set.

### Temporal validation

In temporal (out-of-time) validation—training on cycles performed in 2019–2022 (n=6,166; CLBR 36.2%) and testing on cycles performed in 2023–2024 (n=1,607; CLBR 28.7%)—the Gradient Boosting model retained meaningful discriminatory performance (AUC 0.712, 95% CI 0.686–0.737; accuracy 0.674; sensitivity 0.610; specificity 0.700; Brier score 0.196). The modest AUC attenuation relative to the random-split test set (0.762) is consistent with the more stringent nature of chronological validation, in which the test cohort differs from the training cohort in calendar time as well as in case mix. Predicted probabilities remained well-calibrated to contemporaneous observed rates, demonstrating that the model transports to a chronologically later cohort rather than overfitting to the training epoch (**Supplementary Figure S1**). Detailed temporal validation metrics are provided in **Supplementary Table S1**.

### Benchmark comparison: separating algorithm from information

On the test cases common to all three models (n=2,217), the McLernon-style logistic regression—incorporating female age, treatment cycle number, infertility type, and parity—achieved an AUC of 0.691 (95% CI 0.669–0.713), consistent with the 0.63–0.68 range reported in recent systematic reviews of existing CLBR prediction models. The GBDT Top-8 model achieved an AUC of 0.761 (95% CI 0.741–0.781), an absolute improvement of 0.071 (DeLong Z = 7.59, p = 3.1×10^-^¹^4^), with continuous NRI 0.629 (95% CI 0.550–0.712) and IDI 0.105 (95% CI 0.090–0.121). The information-matched comparison, however, localizes the source of this gain. A gradient-boosting model restricted to pre-treatment variables achieved an AUC of 0.693 (95% CI 0.671–0.715), which was indistinguishable from the logistic benchmark built on the same class of information (absolute difference 0.002; DeLong Z = 0.36, p = 0.72). By contrast, adding embryological information to that gradient-boosting model raised the AUC by 0.068 (0.761 vs 0.693; DeLong Z = 7.80, p = 6.4×10^-^¹^5^; NRI 0.536, 95% CI 0.458–0.619). The improvement over established frameworks is therefore attributable to the inclusion of embryological information rather than to the flexibility of the learning algorithm (**Supplementary Table S2**, **Supplementary Figure S2**).

### Subgroup calibration by female age

Age-stratified calibration analysis demonstrated consistent discriminatory performance and satisfactory calibration across all clinical subgroups (**Supplementary Table S3**, **Supplementary Figure S3**). AUC values were 0.695 (95% CI 0.668–0.724) for women aged<35 years (n=1,261), 0.719 (0.670–0.766) for 35–37 years (n=446), 0.784 (0.727–0.837) for 38–40 years (n=322), and 0.712 (0.604–0.805) for >40 years (n=303); the four strata together comprise the complete test set. Calibration was close across every stratum, with the mean predicted probability deviating from the observed CLBR by at most 0.018 (35–37 years: 0.330 vs 0.312). Agreement was particularly good in the two oldest strata, where the mean predicted probability was 0.227 versus an observed 0.217 at 38–40 years and 0.083 versus 0.092 above 40 years, supporting reliable individualized counselling in these clinically important groups. Brier scores ranged from 0.081 (>40 years) to 0.219 (<35 years), consistent with the lower event rate in older women.

### Dynamic sequential prediction across decision points

Reconstructing the model at three sequential clinical decision points revealed that predictive accuracy accrued non-uniformly across the treatment pathway (**Supplementary Table S4**, **Supplementary Figure S4** and **S6**). The pre-treatment model (Stage 1; 10 variables) achieved a test-set AUC of 0.693 (95% CI 0.672-0.715). Adding endocrine and ovarian-response parameters at the post-retrieval stage (Stage 2; 15 variables) yielded only a marginal improvement (AUC 0.701; 95% CI 0.679-0.724). In contrast, the post-transfer model (Stage 3; the eight selected predictors, which include embryo quality, developmental stage, and transfer parameters) produced a substantial gain in discrimination (AUC 0.762; 95% CI 0.743-0.781). Stage-specific SHAP analyses (**Supplementary Figure S5**) confirmed that embryo quality dominated the Stage 3 feature-importance ranking. However, the stage-specific models used different predictor sets and available-case test sample sizes (2,217, 2,154, and 2,332, respectively); therefore, the AUC differences should be interpreted as stage-specific associations rather than as an isolated effect attributable solely to embryological information.

## Discussion

### Principal findings

This study developed and validated interpretable machine-learning models to predict cumulative live birth in ART. Among six candidate algorithms, gradient boosting achieved the highest discrimination on the random-split test set (AUC 0.762; 95% CI 0.743–0.781) and remained robust under temporal validation on a chronologically later 2023–2024 cohort (AUC 0.712). It also substantially outperformed a McLernon-style logistic-regression benchmark (0.761 vs 0.691; DeLong p<0.0001; NRI 0.629); however, an information-matched comparison showed this advantage did not arise from the learning algorithm itself, since a gradient-boosting model given only pre-treatment variables performed no better than the benchmark (0.693 vs 0.691; p=0.72), whereas adding embryological information raised discrimination by 0.068.

SHAP analyses indicated that, at the individual level, female age was the most influential predictor (mean |SHAP| = 0.494), followed by embryo quality (0.451). While embryo quality provided the strongest population-level discrimination in the gain-based ranking, maternal age exerted a greater impact on patient-specific outcome variability, with the contribution of age turning negative between 37 and 38 years and declining steeply thereafter.

### Comparison with existing literature

Our findings advance beyond previous CLBR prediction models that typically achieved AUC values of 0.63–0.68, as reported in recent systematic reviews (10). The performance of our Gradient Boosting model (AUC 0.762) principally reflects the inclusion of embryological information, which is unavailable to pre-treatment frameworks, together with systematic feature selection, complete data capture across all screened cycles, and rigorous internal validation. As our information-matched comparison shows, it does not reflect an intrinsic advantage of ensemble learning over logistic regression when both are given the same variables.

Several recent investigations have explored machine learning for ART outcome prediction (11), often attributing gains to the ability of flexible algorithms to capture non-linear relationships that logistic regression cannot represent (9). Our information-matched comparison qualifies that interpretation: when the same predictors were supplied to both model classes, gradient boosting conferred no measurable advantage over logistic regression, and the entire performance gain was attributable to the predictors themselves. Reported machine-learning advantages in this literature may therefore partly reflect differences in the variables supplied to each model rather than in algorithmic capacity.

The dominance of female age and embryo quality as predictive factors corroborates reproductive medicine literature while quantifying their relative contributions through interpretable machine learning (2). Notably, our analysis revealed that embryo quality demonstrates a clear stepwise gradient in predictive contribution, with each morphological grade improvement conferring measurable increases in predicted live birth probability. This granular understanding extends beyond binary quality classifications commonly employed in clinical practice.

### Temporal validity and clinical applicability

A key limitation of most published ART prediction models is that training and testing rely on random splits from the same time period, which overstates real-world generalizability. We addressed this through temporal (out-of-time) validation, training on 2019–2022 data and evaluating on 2023–2024 cycles. Discrimination was retained with only modest attenuation relative to the random-split estimate, and predicted probabilities remained aligned with contemporaneous observed rates, supporting the model’s transportability to future patients at the same centre—the setting in which such a tool would actually be deployed.

### Benchmarking against established prognostic tools

Few machine-learning studies in reproductive medicine directly compare their models against the established clinical standard. We constructed a McLernon-style logistic regression using the four core McLernon pre-treatment predictors and, recognising that such a comparison confounds algorithm with information, added a gradient-boosting model restricted to the same pre-treatment variables. This three-way comparison separated the two effects: our full model exceeded the benchmark with substantial reclassification improvement, but the information-matched model did not differ from it. This finding is consistent with the dynamic sequential analysis below and carries a practical implication: efforts to improve CLBR prediction are likely to yield more from expanding the information available at the moment of prediction than from substituting more flexible algorithms for regression.

### Age-stratified calibration and equity considerations

Prediction models that perform well on aggregate metrics may nonetheless exhibit systematic miscalibration in high-risk subgroups, so we evaluated performance within four clinically defined age strata. Discrimination was maintained across all groups (AUC 0.695–0.784), with the highest point estimate in the 38–40 year stratum, although overlapping confidence intervals preclude firm conclusions about differential discrimination. Calibration was close throughout, and the model did not overestimate success in the oldest women (mean predicted probability 0.083 vs observed CLBR 0.092 above age 40) — a property that matters clinically because overestimation could support inappropriate continuation of treatment. These findings support equitable application across the reproductive-age spectrum and the principle that prognostic tools should be validated in the subgroups where accurate counselling matters most.

### The value of information across the treatment pathway

Our dynamic sequential analysis offers a clinically actionable perspective absent from single-timepoint models: by reconstructing the model at three decision points, we quantified how prognostic information accrues across the treatment pathway. The near-absence of improvement from pre-treatment to post-retrieval (AUC 0.693 to 0.701) indicates that endocrine and ovarian-response parameters add limited discriminatory value beyond baseline demographic and ovarian-reserve variables, despite their central role in cycle management. The marked gain at the post-transfer stage (AUC 0.762) was temporally associated with the availability of embryological information, particularly embryo quality. However, because the stage-specific models used different predictor sets and available-case sample sizes, this gain cannot be attributed solely to embryological information. Together with the information-matched benchmark comparison, these findings suggest that the information available at the time of prediction may contribute more to discrimination than the choice between the evaluated algorithms.

### Interpretability versus causality

An important caveat concerns SHAP interpretation. Although SHAP decomposes predictions into transparent, additive feature contributions, these quantify statistical association within the model, not causal effects. Because the data are observational and subject to residual confounding, a feature’s high SHAP importance — such as embryo quality or female age — should not be read as evidence that modifying that variable would causally change live-birth probability, particularly as several predictors here are correlated and jointly determined (see Limitations). The SHAP explanations reported are intended to enhance transparency, auditability, and clinician–patient communication, not to support causal or mechanistic claims; prospective and mechanistic studies remain necessary to establish causal relationships.

Beyond discrimination, the model showed good calibration and positive net benefit across clinically relevant threshold probabilities (0.30–0.40) (28), and its SHAP-based interpretability translates predictions into transparent, patient-specific explanations (16). For example, in a patient with advanced maternal age but high-quality embryos, clinicians can ground individualized counselling in a quantified, feature-level risk explanation rather than in subjective judgment alone.

The eight selected predictors offer practical guidance for streamlining data collection for prediction purposes. Because these variables carry the strongest gain- and SHAP-based signal for cumulative live birth prediction in this model, prioritizing their collection may improve clinical workflow efficiency; this should not be read as evidence that they are the only, or even the principal, independent causal determinants of cumulative live birth, a distinction addressed above. Sensitivity analysis confirms that restricting the model to these eight variables maintains robust predictive performance, supporting both clinical feasibility and predictive accuracy without unnecessary complexity.

### Methodological strengths

Several methodological strengths distinguish this investigation. First, systematic comparison of multiple algorithms under identical training/testing protocols enabled unbiased algorithm selection. Second, stratified sampling and cross-validation with bootstrap-derived confidence intervals provided robust internal validation. Third, feature selection was conducted without deleting any record, and the resulting predictors were complete in every screened cycle, so all 7,773 cycles entered the analysis without selection bias from listwise deletion. Fourth, the benchmark comparison held information constant in one arm, distinguishing the algorithm’s contribution from the predictors’ contribution — a distinction rarely drawn in this literature.

The sensitivity analyses reinforce these conclusions: because the eight predictors were complete in all cycles, the primary analysis was unaffected by missing-data assumptions, and in the extended twelve-predictor analysis, complete-case and imputed models differed by only 0.002 in AUC. Discrimination peaked at eight features and did not improve with ten or twelve, validating our parsimonious modeling approach.

### Limitations and future directions

Several limitations warrant consideration. First, the retrospective single-center design may limit generalizability; although temporal validation on the 2023–2024 cohort showed performance was maintained across a chronological boundary, external validation at other centers remains necessary before wider implementation. Second, the conservative cumulative live birth rate treats untransferred cryopreserved embryos as not achieving live birth, and cycles near the end of the study period had shorter follow-up, so cumulative live birth may be underestimated in the most recent cycles. Third, the dataset lacked a patient-level identifier, so cycles from the same woman could not be linked or analyzed with clustering-aware methods (e.g., generalized estimating equations); the resulting non-independence, most relevant for women with repeated cycles, may render standard errors slightly optimistic, and its magnitude could not be quantified with the data available. Fourth, cycles without embryo transfer or with undefined outcomes were excluded at eligibility screening (Materials and Methods); the pre-eligibility log was not retained, so this exclusion cannot be quantified, but such cycles often reflect the poorest-prognosis cases and their omission likely restricts applicability to women who reach embryo transfer.

Fifth, embryo quality was recorded as a composite quality-and-count index rather than a per-embryo morphology grade (Materials and Methods) and is likely correlated with maternal age, since younger women more often produce embryos of both better morphology and higher euploidy; gain- and SHAP-based importance does not require predictor independence, so this collinearity does not invalidate the prediction goal, but individual contributions attributed to age and embryo quality should not be read as independent causal effects. Sixth, embryo euploidy status was not captured; PGT-A results correlate only imperfectly with morphological grading and are among the strongest known predictors of live birth in older women, so the model reflects morphology- rather than ploidy-based information, which should be considered when counselling older patients. Finally, the AUC of 0.762 leaves room for improvement; additional modalities such as time-lapse morphokinetics, PGT-A, or endometrial receptivity markers may help, and our benchmark comparison suggests such gains are more likely to come from richer information than from more complex algorithms.

Future research should explore several promising directions. Deep learning approaches applied to embryo images could capture morphological features beyond conventional grading systems. Incorporation of partner-specific factors, particularly advanced paternal age and sperm DNA fragmentation, may improve prediction accuracy. Multi-center collaborative efforts using federated learning could develop models that maintain privacy while benefiting from diverse populations.

## Conclusion

This study developed and validated an interpretable machine-learning framework to predict cumulative live birth in ART. While preserving clinical interpretability through SHAP analysis, the framework delivered strong predictive performance. The gradient boosting model demonstrated robust discrimination on a random-split test set (AUC 0.762) and under chronological validation (AUC 0.712), with good calibration across the reproductive-age spectrum. Its advantage over an established pre-treatment benchmark was attributable to the inclusion of embryological information rather than to the learning algorithm itself: precise estimation of cumulative live birth requires embryological data that become available only after oocyte retrieval and embryo development, rather than depending on which machine-learning algorithm is used to model that information.

## Data Availability

The datasets are not publicly available due to patient confidentiality, but may be obtained from the corresponding author on reasonable request, subject to institutional and ethical approval.

## Supplementary material

Supplementary Table 1 Temporal validation performance of the gradient-boosting model (trained on 2019–2022 cycles, n=6,166; tested on 2023–2024 cycles, n=1,607). Reported metrics include AUC with 95% confidence interval, accuracy, sensitivity, specificity, and Brier score. For reference, the random-split test set yielded an AUC of 0.762.

Supplementary Table 2 Benchmark Comparison: Head-to-Head Results. AUC with 95% confidence intervals, Brier score, DeLong Z-statistic and p-value, continuous net reclassification improvement (NRI) with its event and non-event components, and integrated discrimination improvement (IDI), each with percentile 95% confidence intervals from 2,000 bootstrap resamples, for three models evaluated on the test cases common to all comparisons (n=2,217): the McLernon-style logistic regression benchmark, an information-matched gradient-boosting model restricted to pre-treatment variables, and the proposed GBDT Top-8 model. Confidence intervals for AUC differences are based on DeLong’s method for correlated ROC curves.

Supplementary Table 3 Age-Stratified Model Performance. For each female-age stratum (<35, 35–37, 38–40, >40 years), the table reports sample size, the observed range of ages contributing to the stratum, observed CLBR, mean predicted probability, AUC with bootstrap 95% confidence intervals, and Brier score. Strata are mutually exclusive and together comprise the complete test set (n=2,332).
