# Supplementary material for: Development and validation of interpretable machine learning models for predicting cumulative live birth rates in assisted reproductive technology: a SHAP-based analysis
Source: Front Endocrinol (Lausanne). 2026 Oct 7;17:1949824. doi: 10.3389/fendo.2026.1949824 (PMC13683200; doi:10.3389/fendo.2026.1949824)
Supplement: Supplementary file 1 [file Table1.docx]

**Supplementary Materials**

**Supplementary Table S1. Temporal Validation Performance (Trained on 2019–2022, Tested on 2023–2024).**

| Cohort | N | CLBR rate | AUC | 95% CI | Accuracy | Sensitivity | Specificity | Brier |
| --- | --- | --- | --- | --- | --- | --- | --- | --- |
| Training (2019–2022) | 6,166 | 0.362 | — | — | — | — | — | — |
| Temporal test (2023–2024) | 1,607 | 0.287 | 0.712 | 0.686–0.737 | 0.674 | 0.610 | 0.700 | 0.196 |

*For reference, the random-split test set yielded an AUC of 0.762. Because all eight model predictors were complete in every cycle, the temporal analysis used the same 7,773 cycles as the primary analysis, partitioned chronologically rather than at random. The modest attenuation under temporal validation is consistent with the more stringent nature of chronological validation, in which the test cohort differs from the training cohort in calendar time as well as in case mix. CLBR, cumulative live birth rate; AUC, area under the receiver operating characteristic curve.*

**Supplementary Table S2. Benchmark Comparison Separating the Contribution of the Algorithm from That of the Available Information (Test Set, n = 2,217).**

| Model | AUC | 95% CI | Accuracy | Sensitivity | Specificity | PPV | NPV | Brier |
| --- | --- | --- | --- | --- | --- | --- | --- | --- |
| McLernon-style LR (pre-treatment) | 0.691 | 0.669–0.713 | 0.664 | 0.281 | 0.869 | 0.536 | 0.692 | 0.206 |
| GBDT, pre-treatment only (information-matched) | 0.693 | 0.671–0.715 | 0.667 | 0.391 | 0.815 | 0.532 | 0.713 | 0.205 |
| GBDT Top-8 (proposed) | 0.761 | 0.741–0.781 | 0.710 | 0.565 | 0.787 | 0.588 | 0.771 | 0.185 |

*All three models were evaluated on the test cases common to every comparison (n = 2,217). GBDT Top-8 versus McLernon-style LR: ΔAUC = +0.071, DeLong Z = 7.59, p = 3.1×10⁻¹⁴; continuous NRI = 0.629 (95% CI 0.550–0.712); IDI = 0.105 (95% CI 0.090–0.121). Information-matched GBDT versus McLernon-style LR: ΔAUC = +0.002, DeLong Z = 0.36, p = 0.72; NRI = 0.210 (95% CI 0.121–0.298). GBDT Top-8 versus information-matched GBDT: ΔAUC = +0.068, DeLong Z = 7.80, p = 6.4×10⁻¹⁵; NRI = 0.536 (95% CI 0.458–0.619). Because the pre-treatment GBDT did not outperform the logistic benchmark while the Top-8 model did, the improvement is attributable to the inclusion of embryological information rather than to the learning algorithm. The McLernon-style model used female age, treatment cycle number, infertility type, and parity. DeLong tests account for correlation between ROC curves estimated on the same cases; NRI and IDI confidence intervals are percentile intervals from 2,000 bootstrap resamples. PPV, positive predictive value; NPV, negative predictive value.*

**Supplementary Table S3. Age-Stratified Performance of the Gradient Boosting Model (Test Set, n = 2,332).**

| Age group (years) | N | Observed age range | Observed CLBR | Mean predicted prob. | AUC | 95% CI | Brier |
| --- | --- | --- | --- | --- | --- | --- | --- |
| <35 | 1,261 | 20–34 | 0.453 | 0.455 | 0.695 | 0.668–0.724 | 0.219 |
| 35–37 | 446 | 35–37 | 0.312 | 0.330 | 0.719 | 0.670–0.766 | 0.188 |
| 38–40 | 322 | 38–40 | 0.217 | 0.227 | 0.784 | 0.727–0.837 | 0.142 |
| >40 | 303 | 41–49 | 0.092 | 0.083 | 0.712 | 0.604–0.805 | 0.081 |

*Strata are mutually exclusive and together comprise the complete test set. The observed age range is reported for each stratum to document the boundaries applied. AUC 95% confidence intervals were derived from 2,000 bootstrap resamples within each stratum; intervals overlap across strata, so differences in discrimination between age groups should not be over-interpreted. The mean predicted probability deviated from the observed CLBR by at most 0.018 in any stratum, and the model did not overestimate success in women older than 40 years.*

**Supplementary Table S4. Dynamic Sequential Prediction Performance Across Three Clinical Decision Points.**

| Stage | Variables | CV AUC | Test n | Test AUC | 95% CI | Accuracy | Sensitivity | Specificity | Brier |
| --- | --- | --- | --- | --- | --- | --- | --- | --- | --- |
| Stage 1 (Pre-treatment) | 10 | 0.709±0.019 | 2,217 | 0.693 | 0.672–0.715 | 0.667 | 0.391 | 0.815 | 0.205 |
| Stage 2 (Post-retrieval) | 15 | 0.720±0.020 | 2,154 | 0.701 | 0.679–0.724 | 0.675 | 0.412 | 0.815 | 0.203 |
| Stage 3 (Post-transfer) | 8 | 0.786±0.024 | 2,332 | 0.762 | 0.743–0.781 | 0.710 | 0.556 | 0.791 | 0.185 |

*Each stage-specific model used the identical data partition and gradient-boosting configuration as the primary analysis. Test-set size differs between stages because some endocrine and ovarian-response variables available at Stages 1 and 2 contain missing values, whereas the eight Stage 3 predictors were complete in every cycle. Predictive accuracy accrued non-uniformly: the gain from Stage 1 to Stage 2 was marginal (+0.008), whereas the gain at Stage 3, when embryological information became available, was substantial (+0.061).*

**Supplementary Figure Legends**

**Figure S1. Temporal Validation ROC Curve. Receiver operating characteristic curve for the Gradient Boosting model evaluated on the temporal holdout cohort (2023–2024, n = 1,607) after training on 2019–2022 data (n = 6,166). AUC = 0.712 (95% CI 0.686–0.737). The shaded area indicates bootstrap 95% confidence intervals.**

**Figure S2. Benchmark Comparison ROC Curves. ROC curves for the McLernon-style logistic regression (AUC 0.691), an information-matched gradient-boosting model restricted to pre-treatment variables (AUC 0.693), and the GBDT Top-8 model (AUC 0.761), all evaluated on the test cases common to the three models (n = 2,217). DeLong tests: Top-8 versus logistic benchmark, Z = 7.59, p = 3.1×10⁻¹⁴; information-matched GBDT versus logistic benchmark, Z = 0.36, p = 0.72.**

**Figure S3. Age-Stratified Calibration Curves.** Calibration curves for the Gradient Boosting model stratified by female age (<35, 35–37, 38–40, >40 years). Each panel plots mean predicted probability against observed CLBR rate; the dashed diagonal represents perfect calibration.

**Figure S4. Progressive Gain in Predictive Accuracy Across Clinical Decision Points. Test-set AUC of the model reconstructed at three sequential stages: pre-treatment (0.693), post-retrieval (0.701), and post-transfer (0.762). The marked gain at the post-transfer stage reflects the incorporation of embryological information.**

**Figure S5. Stage-Specific SHAP Importance.** SHAP mean-absolute-value feature-importance rankings for the pre-treatment (A), post-retrieval (B), and post-transfer (C) models, demonstrating the dominant contribution of embryo quality once it becomes available.

**Figure S6. Sequential Prediction ROC Curves.** ROC curves for the three stage-specific models on the independent test set.
